# Synthetic Ligands of Olfactory Binding Proteins Modulate Aggregation Response of Asian Citrus Psyllid in the Presence of Host-Plant Volatiles

**DOI:** 10.3389/fpls.2018.01891

**Published:** 2018-12-20

**Authors:** Joseph M. Patt, William G. Meikle, Randall P. Niedz, Daniel Woods

**Affiliations:** ^1^USDA-ARS U.S. Horticultural Research Laboratory, Fort Pierce, FL, United States; ^2^USDA-ARS Carl Hayden Bee Research Center, Tucson, AZ, United States; ^3^Inscent, Inc., Irvine, CA, United States

**Keywords:** citrus greening, insect attractant, *Diaphorina citri*, ligand, chemosensory binding protein, volatile, semiochemical

## Abstract

There is interest in using ligands of chemosensory binding proteins (CBP) to augment an insect’s responsiveness to chemosensory cues. We showed previously that combining a synthetic ligand of a CBP with limonene, a common citrus volatile, enhanced the probing response of Asian citrus psyllid (*Diaphorina* *citri*). Here, we determined whether synthetic compounds, which were ligands of *D*. *citri* olfactory binding protein (OBP) DCSAP4, influenced the settling and aggregation levels of psyllids on young citrus shoots. The test ligands and Cmac scent were dispensed from a droplet of an emulsified wax product (SPLAT) placed on the bottom of each vial. The shoots were presented: (1) alone (shoot + blank SPLAT), (2) with a mixture of citrus volatiles (“Cmac scent”) (shoot + SPLAT with Cmac scent), or (3) with different concentrations of test ligands (shoot + SPLAT with test ligand at concentration 1, shoot + SPLAT with test ligand at concentration 2, etc.). Depending on the availability of test ligands, sprigs, and psyllids, each test included from two to four replicates of each treatment (i.e., shoot only, shoot + Cmac scent, shoot + test ligand at concentration 1, shoot + test ligand at concentration 2, etc.); only a single test ligand was presented in each test. For each test, 200 *D. citri* were released in the test area and the numbers of psyllids on each sprig were counted 24 h later. Sprigs with ≥7 psyllids were considered to be an aggregation. A total of seven ligands were tested individually. Four of the ligands (654, 717, 784, and 861) modulated psyllid settling and aggregation response, causing greater settling and aggregation to sprigs presented with the Cmac scent than to those sprigs with blank SPLAT. Presentation of one of the ligands (019) resulted in an opposite effect in which psyllid settling and aggregation levels were lower on sprigs with Cmac scent than on those with blank SPLAT. There were no differences in settling levels in the different treatment vials in the Ligand 905 experiment. In the Ligand 937 experiment, settling levels did not vary significantly between treatment vials although settling levels were relatively high in all treatment vials and there was a significant treatment effect. Increased settling and aggregation levels were largely not observed with in the vials with only the test ligands, and there was little effect of ligand concentration on psyllid response levels. This suggests that the test ligands themselves did not attract the psyllids but rather modulated the psyllid’s response to the Cmac scent. The results suggest that synthetic ligands of *D*. *citri* CBPs can be used to increase the effectiveness of citrus scent lures used to attract psyllids to monitoring traps and attract and kill devices.

## Introduction

The Asian citrus psyllid, *Diaphorina citri* (Kuwayama) (Hemiptera: Psyllidae) is the vector of *Candidatus* Liberibacter asiaticus, the causal agent of citrus greening disease or Huanglongbing (Halbert and Manjunath, 2004; Grafton-Cardwell et al., 2013; Hall et al., 2013). Citrus greening is the most devastating disease of citrus in the world today and has resulted in the loss of hundreds of thousands of hectares of citrus groves and billions of dollars in productivity (Bové, 2006; Gottwald, 2010; Wang and Trivedi, 2013; Spreen et al., 2014). ACP inhabits citrus trees in both commercial groves and residential areas and can move over large distances in a relatively short time (Boina et al., 2009; Tiwari et al., 2010; Martini et al., 2014; Lewis-Rosenblum et al., 2015).

Detection and monitoring efforts currently rely on tap sampling, direct visual counts, and yellow sticky card traps (Hall et al., 2007;Sétamou et al., 2008; Qureshi et al., 2009; Hall and Hentz, 2010; Hall et al., 2010); the former are labor intensive while the latter may provide inconsistent results. There is a great need for more reliable monitoring methods to effectively detect psyllid infiltration into citrus producing areas, monitor their population densities, and track the efficacy of control measures. *D*. *citri* is attracted to the young shoots of *Citrus* and closely related genera. The tender shoots are the only place where it mates, oviposits, and develops (Halbert and Manjunath, 2004; Grafton-Cardwell et al., 2013; Hall et al., 2013; Cifuentes-Arenas et al., 2018). The stimuli involved in host plant location and selection behavior by *D*. *citri* encompass a combination of visual cues (Wenninger et al., 2009b; Sétamou et al., 2014; Paris et al., 2015, 2017a,b), constitutive and induced host plant volatiles (Patt and Sétamou, 2010; Mann et al., 2012; Patt et al., 2014; Stockton et al., 2016; Patt et al., 2018), and conspecifics’ pheromones (Wenninger et al., 2008; Martini et al., 2014; Stockton et al., 2017) and vibrational signals (Wenninger et al., 2009a; Rohde et al., 2013). Determining the identity of these stimuli is necessary to develop effective traps for detecting the presence of adult psyllids and monitoring their population density.

While visual cues are probably the primary long distance attractant for *D*. *citri*, olfactory cues could make an important contribution at close range to trap effectiveness (Patt and Sétamou, 2010). However, the complexity and variation inherent in citrus foliar volatiles makes it difficult to formulate scent lure mixtures with the composition, proportion, and concentration needed to function effectively.

There is potential for using ligands of olfactory binding proteins (OBPs) as surrogate scent attractants, repellents, or confusants, or to augment an insect’s responsiveness to natural olfactory cues (Zhou et al., 2010). Although the *D*. *citri* antenna has relatively few sensillar hairs (Onagbola et al., 2008), transcriptome analysis revealed that these structures contain numerous olfactory receptors, OBPs, and associated proteins (Wu et al., 2016) and electrophysiological studies demonstrated response to a large number of volatile compounds from several chemical classes (Coutinho-Abreu et al., 2014a,b). To evaluate *D*. *citri* behavioral response to synthetic compounds identified as putative ligands of antennal OBPs, Patt et al. (2013) measured the number of salivary sheaths deposited in artificial midribs made from an emulsified wax, commonly called SPLAT (ISCA Tech, Inc.). They found that probing level was significantly higher in midribs containing a mixture of limonene and a particular synthetic ligand than in midribs containing either limonene or the ligand alone. This result indicated that the addition of an OBP ligand could enhance the responsiveness of *D*. *citri* to naturally occurring citrus foliar volatiles.

In this study, we used a settling vial assay to determine whether the presence of putative ligands of *D*. *citri* OBPs influenced the psyllid’s settling and aggregation levels on young citrus shoots, both when the shoots were presented alone or in combination with a mixture of citrus volatiles.

## Materials and Methods

### Study Plants and Insects

Psyllids used in the experiments were obtained from a colony of *D. citri* maintained at the USDA-ARS laboratory in Fort Pierce, FL, United States. Young sprigs were obtained from potted *Citrus macrophylla* (Wester) raised in a greenhouse at the same location.

### Selection of Novel Synthetic Ligands

The synthetic compounds were initially identified as novel ligands of OBPs using the Attenu assay, a proprietary, high throughput assay developed by Inscent, Inc. Attenu uses fluorescence quenching to identify the level of interaction between OBPs and potential ligands. When a putative ligand displaces a fluorescent marker from the binding pocket of an OBP, the reduction in fluorescence indicates that the test ligand reacted with the OBP. OBPs isolated from *D*. *citri* were used as screening targets to identify potential synthetic ligands. The most reactive compounds were then screened to measure influence on psyllid probing behavior (Patt et al., 2013). One synthetic compound, identification number 5276937, elicited the highest level of probing response by the psyllids. In the Attenu assay, this synthetic ligand reacted with OBP DcSAP4. All of the ligands tested in the present study were structurally related to Ligand 5276937. Because the ligands are proprietary, their structures are not shown here.

The test ligands used in the present study each had an identifying serial number, which was abbreviated to the last three digits for recording the results. They were identified as follows: 5267019 (=“019”), 64288654 (=“654”), 9325717 (=“717”), 5649784 (=“784”), 5655861 (=“861”), and 6428905 (=“905”). Ligand 5276937 (=“937”) was included in the present study because the other test ligands are structurally related to it and it was previously shown to increase behavioral responsiveness of *D*. *citri* (Patt unpublished data).

### Behavioral Assay

A modification of the settling assay used in Hall et al. (2015) and Patt et al. (2018) was used to evaluate psyllid settling and aggregation responses on citrus sprigs in the presence of either test ligands or synthetic host plant volatiles. The assay consisted of presenting the psyllids with a series of settling vials, each containing a citrus sprig and a scent dispenser consisting of a droplet of an emulsified wax product (SPLAT, ISCA Tech, Inc.) (Patt et al., 2011). The SPLAT droplet was placed in the center of a filter paper disk inserted into the bottom of the test vial and contained an aliquot of either a test ligand or an artificial citrus scent mixture. A blank SPLAT droplet was used in the negative control treatment.

**FIGURE 1:**
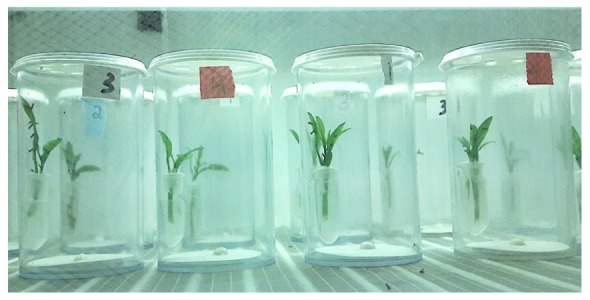
Settling vial assay. Each vial has a droplet of SPLAT at the bottom, which functions as a test scent or OBP ligand dispenser. Note the clumped distribution of psyllid distribution within the vials.

A young sprig, ca. 2.5 cm long, was placed in an individual vial (25 dram clear-plastic vials, 39 mm diameter × 85 mm high (BioQuip Products, Inc., Gardenia, CA, United States) and capped with a white snap-on plastic lid. The sprigs were excised from potted *C. macrophylla* because the psyllids were reared on this plant and *D. citri* responds behaviorally more strongly to its natal host plant than to novel host plants (Patt et al., 2014; Stockton et al., 2016). The cut ends of the sprig was placed into a 1.3 mL Eppendorf vial filled with tap water; the Eppendorf tube was held in place with plastic holders glued to the inside of the vial. The vial cap had a series of 6 mm diameter holes to permit psyllid entry into the vial (Figure 1). Once inside the vial, psyllids strongly tended to feed and remain on the sprigs. The test ligands were dispensed *via* a 100 μL droplet of SPLAT applied to the center of a filter paper disk placed on the bottom of the test vial (Figure 1). White-colored SPLAT was used to preclude any confounding effects from visual attraction to the test odor source.

The vials were placed in an incubator fitted with fine screening to confine the psyllids in an area ca. 20.5 cm high × 67.0 cm wide × 41.5 long. The vials were arranged in two rows directly underneath fluorescent lights positioned 13.5 cm above the vial caps. The positions of the vials were randomized to prevent spatial bias. The lights were programed for a 16-h light: 8-h dark cycle. Two hundred adult psyllids, 5–10 days old (post-eclosion), were released into each incubator at the start of the test. After 24 h, the vials were sealed and placed in a freezer for 2 h. After freezing, the numbers of psyllids in each vial were counted.

Limited amounts of ligands were available for testing. When 100 mg of test ligand was available, the following concentrations were tested: 3-, 9-, 30-, and 90 mg OBP ligand/10 mL SPLAT. This concentration series was used for ligands 019, 654, and 937 (Table 1). When 50 mg of test ligand was available, concentrations of 9- and 30 mg OBP ligand/10 mL SPLAT were tested. This concentration series was used for ligands 717, 784, 861, and 905 (Table 1).

**Table 1:** Design of individual experiments showing treatments included in each, number of vials per treatment per each test, and the total number of vials used in each treatment in each experiment.

	Treatments included in experiment								
Experiment	Blank SPLAT	3 μL Cmac scent	9 μL Cmac scent	3 mg test ligand	9 mg test ligand	30 mg test ligand	90 mg test ligand	Number of vials per treatment per test	Total number of vials tested per treatment
No ligand	X	X						4	64
937	X	X	X	X	X	X	X	2	27
019	X	X		X	X	X	X	2	40
654	X	X		X	X	X	X	2	48
717	X	X			X	X		4	48
784	X	X			X	X		4	72
861	X	X			X	X		4	60
905	X	X			X	X		4	48


Included in the test design were a negative control (citrus sprig + 100 μL blank SPLAT droplet) and a positive control where a citrus scent was added (citrus sprig + 100 μL SPLAT droplet with artificial citrus scent), since preliminary work indicated that the citrus scent was an attractant to *D*. *citri*. The composition of the artificial citrus scent (“Cmac” scent) was based on the most prevalent volatiles emitted from the young shoots of *C*. *macrophylla*. It contained a mixture of synthetic volatiles in the following proportions: 6.0 limonene: 3.6 citral: 1.2 β-ocimene: 1.0 β-caryophyllene: 1.0 terpinene: 0.6 linalool: 0.02 methyl salicylate.

The test with Ligand 937 was the first to be conducted. Its design included two concentrations of Cmac scent, 3- and 9 μL/10 mL SPLAT; these were compared to determine an optimal concentration of Cmac scent to use in tests with the other ligands. Since psyllid response was stronger to the 3-μL concentration of Cmac scent, only this concentration was used in subsequent tests.

In the Ligand 937 tests, there were seven treatments; each treatment vial was replicated twice in each incubator (14 vials/incubator). In the tests with ligands 019 and 654, there were six treatments; each treatment vial was replicated twice in each incubator (12 vials/incubator). In the tests with ligands 717, 784, 861, and 905, there were four treatments; each treatment was replicated four times (16 vials/incubator). Depending on the availability of sprigs and psyllids, three to four tests were conducted simultaneously in separate incubators for each 2-day experiment, resulting in 12–28 replicate experiments per ligand. Only a single ligand was tested simultaneously in the different incubators, and only a single ligand was tested during each complete experiment.

A separate test was conducted to evaluate psyllid settling levels in vials when no ligands were present. This test consisted of vials with a citrus sprig + a SPLAT droplet with Cmac scent and vials with a citrus sprig + a blank SPLAT droplet. A ratio of one Cmac vial: three blank SPLAT vials, with a total of 16 vials/incubator, was used to maintain a vial placement and density pattern that was similar to those used in the test ligands experiments.

### Distribution Induces and Statistical Analysis

Data on psyllid distribution in incubators were analyzed as follows: each vial in the incubator was considered an experimental unit, or sample. Each incubator trial was considered a separate replicate. Mixed-model ANOVA was conducted for each test ligand, with treatment as a fixed effect and trial number (replicate) as a random effect (SAS version 9.4). *Post hoc* contrasts with a Bonferonni correction for multiple comparisons was conducted for all analyses with significant treatment effects using the least squares means test.

To ascertain the level of psyllid aggregation in the different vial treatments, the index corresponding to randomness (*D*_P_), and the Morisita index (*I*_m_) were calculated for each treatment (Hurlbert, 1990; Nansen et al., 2006; Mankin et al., 2014). The *D*_P_ is based on the degree of overlap between the observed and a Poisson (random) distribution. The value of *D*_P_ varies between 0 and 1, with 0 equal to 100% concordance with a Poisson distribution whereas values approaching 1 indicate an aggregated distribution (Hurlbert, 1990). The *I*_m_ measured how many times more likely it is that two randomly selected psyllids will be from the same vial than it would be if the psyllids that settled during the 24-h period (the functional population) were distributed at random, with *I*_m_ equal to 1.0 for random distributions (Hurlbert, 1990). These indices were then used as response variables in ANOVA tests comparing the ligands.

To determine the degree of the randomness in the distribution of psyllids within the different treatment vials, Poisson distributions were calculated for each treatment in each ligand test. The goodness of fit of the Poisson distribution to the observed data was then tested with the chi-square test (Zar, 1999).

As an additional measure of psyllid aggregation in each treatment, we determined the percentage of the total number of psyllids that arose from vials with ≥7 psyllids. A frequency of ≥7 psyllids per vial was selected as representing an aggregation because the presence of seven or more psyllids within a vial appeared to the observer to be definitively “crowded” (Figure 1). For each treatment within each test ligand experiment, the numbers of psyllids in vials with ≥7 psyllids were subjected to planned paired comparisons with the chi-square test (Zar, 1999) with α = 0.0083. For these comparisons, the number of psyllids in vials with ≥7 psyllids in the blank SPLAT treatment was used as the expected frequency in the experiment-wide comparisons. The reason for using the blank SPLAT value as the expected frequency was that the frequency of psyllids that settled on citrus sprigs in control vials with no additional olfactory stimulus present within the vial was considered to represent a baseline level of settling behavior.

## Results

The *D*_p_ index shows the degree of overlap of the observed distribution with the Poisson distribution of the same population mean (Hurlbert, 1990). A *D*_p_ = 0 means that the population has a perfect Poisson distribution within the study area, while a *D*_p_ = 1 means that all the individuals are highly aggregated in a particular portion of the study area. In our study, the vials represented the study area and the values of the *D*_P_ index ranged from 0.315 to 0.433. These values indicated that psyllid distributions in the vials were non-random in all cases (Table 2). *D*_P_ values were not different among test ligands (*F*_7,139_ = 2.26; *P* = 0.330).

**Table 2:** Indices used to ascertain aggregation response of *D*. *citri* in settling vial assays.

Ligand	*D*_P_ index	*I*_m_ index
None	0.380 (0.03)^a^	1.93 (0.18)^a^
019	0.426 (0.03)^a^	1.33 (0.06)^bc^
654	0.433 (0.03)^ab^	1.58 (0.08)^ac^
717	0.366 (0.04)^a^	1.50 (0.13)^ac^
784	0.391 (0.03)^a^	1.53 (0.10)^ac^
861	0.341 (0.03)^a^	1.53 (0.10)^ac^
905	0.385 (0.04)^a^	1.47 (0.15)^ac^
937	0.315 (0.02)^ac^	1.46 (0.07)^bc^


*D_P_, index corresponding to randomness; *I*_*m*_, Morisita index. Values shown are mean ± SEM. Means with different letters in the same column are different, *P* ≤ 0.03.*

The *I*_m_ index indicates how many times more likely it is that two randomly selected psyllids will be from the same vial than if they were randomly distributed. For example, if *I*_m_ = 1.5 then the probability of those two randomly selected individuals are from the same quadrat is 50% greater than it would be in the case of a random distribution. In the present study, *I*_m_ values ranged from 1.33 to 1.93 (Table 2), showing that psyllids were 20–40% more clumped than they would be if their distributions were random. *I*_m_ values among test ligands were different (*F*_7,139_ = 2.43; *P* = 0.0225), with the largest difference in *I*_m_ values observed between test vials containing Ligand 019 test and the control test with no ligands (Table 2).

**FIGURE 2:**
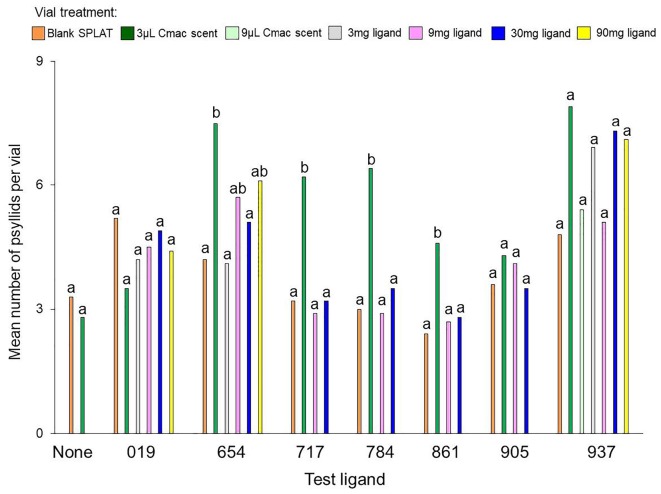
Mean number of psyllids per vial in each separate experiment with the test ligands and the blank SPLAT control. Bars with different letters within the same test ligand are different at *P* ≤ 0.05.

**FIGURE 3:**
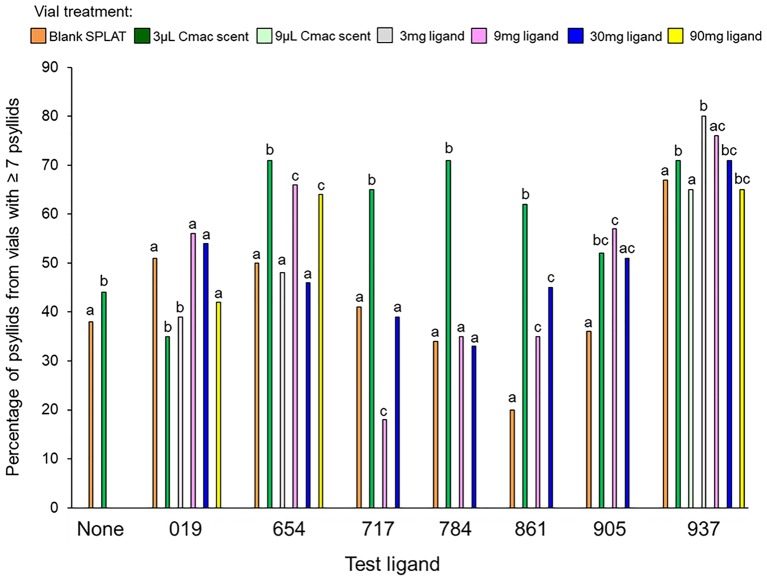
Percentage of psyllids from vials with ≥7 psyllids in each treatment. Bars with different letters within the same test ligand group are different at *P* ≤ 0.05.

In the control test that evaluated psyllid settling levels in the absence of test ligands, similar numbers of psyllids settled in the Cmac scent treatment as in the blank SPLAT treatment (Table 3 and Figure 2). Because psyllid settling levels were similar between the blank SPLAT and Cmac scent treatment, the addition of the Cmac scent did not appear to increase psyllid attraction *per se*. However, the Cmac scent treatment had a greater percentage of psyllids from vials with ≥7 psyllids than did the vials with blank SPLAT (Figure 3), suggesting that the presence of Cmac scent had some influence on psyllid aggregation behavior. Neither of the observed distributions in blank SPLAT control and Cmac scent vials fit the Poisson distributions (Figure A1 in Supplementary Material), demonstrating that the distributions in both treatments were non-random.

**Table 3:** Comparison of the mean numbers of psyllids per vial between treatments within each ligand test.

Ligand	*F* value	*P*-value
None	0.11	0.7418
019	1.6	0.1631
654	4.86	0.0003
717	11.19	<0.0001
784	16.01	<0.0001
861	5.94	0.0007
905	0.45	0.7174
937	2.13	0.0493


*The F values were obtained by ANOVA; the *P-*value is shown in the right hand column.*

The mean number of psyllids per vial varied between treatments in all of the ligand tests except for Ligands 019 and 905 (Table 3). In tests with Ligands 717, 784, and 861, vials with Cmac scent had greater numbers of psyllids per vial than in the other treatment vials (Figure 2). In Ligand 654 test, the settling level was higher in the Cmac scent treatment than in the blank SPLAT and 30 mg ligand concentration treatments. In tests with Ligands 654, 717, 784, and 861, the percentage of psyllids from vials with ≥7 psyllids was also greatest in the vials with Cmac scent (Figure 3). In the Ligand 784 and 861 tests, none of the observed distributions in the individual treatments matched the Poisson distribution (Figures A2, A3 in Supplementary Material). In the Ligand 717 test, the observed distribution in the Cmac scent treatment did not fit the Poisson distribution while those in the blank SPLAT, 9 and 30 mg ligand treatments were similar to the Poisson distribution (Figure A4 in Supplementary Material). In the Ligand 654 test, only the observed distribution in the 30 mg ligand treatment fit the Poisson distribution (Figure A5 in Supplementary Material).

There was no difference in the mean number of psyllids/vial among the treatments in the tests with ligands 019, 905, and 937 (Table 3). In the Ligand 019 test, numerically fewer psyllids settled in the vials with Cmac scent (Figure 2), while the Cmac scent treatment in this experiment also had the lowest frequency of vials with ≥7 psyllids (Figure 3). Only the observed distribution in the Cmac scent treatment matched the Poisson distribution (Figure A6 in Supplementary Material). In the tests with ligands 905 and 937, similar numbers of psyllids settled in the Cmac and blank SPLAT treatment (Figure 2). However, in both ligands, the Cmac scent treatment had a higher percentage of psyllid from vials with ≥7 psyllids than did the blank SPLAT treatment (Figure 3); and none of observed distributions fit the Poisson distribution (Figures A7, A8 in Supplementary Material).

## Discussion

### Olfactory Stimuli and Psyllid Aggregation

Since the psyllids were reared on *C*. *macrophylla*, it was expected that the Cmac scent would function as an attractant and lure psyllids into the test vials. However, because the settling levels of psyllids were similar in the blank SPLAT and Cmac scent vials, it appears that the Cmac scent did not function as an attractant, i.e., it did not lure more psyllids into the vials with Cmac scent. On the other hand, psyllid aggregation levels were slightly higher in the vials with Cmac scent, indicating that it did have a modest effect on the psyllids’ aggregation behavior. In a previous study that used a settling vial assay, similar results were observed in which the mean numbers of psyllids per vial were similar among control and treatment vials but enhanced psyllid aggregation levels were observed in treatment vials (Patt et al., 2018). The results of these studies with settling vial assays suggest that, while the mean number of psyllids per vial is the primary indicator of psyllid response to a particular test odorant, aggregation level is also an important indicator of a test odorant’s biological activity. The values of the aggregation indices showed that psyllid distribution was clumped in most of the ligand tests. Comparison of the observed frequency distributions with the Poisson distribution also demonstrated that, in most cases, the psyllids’ distribution was non-random. The distribution of *D*. *citri* within citrus trees and groves is aggregated (Tsai et al., 2000; Sétamou et al., 2008; Costa et al., 2010; Hall and Hentz, 2010). Because multiple matings are apparently required for high reproductive output by female psyllids (Wenninger and Hall, 2007), aggregation behavior may play a central role in *D*. *citri* reproduction. If this is true, then olfactory stimuli that promote aggregation behavior may be an important means of increasing the efficacy of traps or attract and kill devices because they would promote psyllid congregation.

### OBP Ligands Modulating the Response of *D. citri* Behavior in Test

Four of the test ligands (654, 717, 784, and 861) enhanced the response of *D*. *citri* to the Cmac scent mixture both in terms of attractiveness and aggregation level. These results concurred with those of Patt et al. (2013), who found that psyllid probing increased when a synthetic OBP ligand was combined with a single citrus volatile, and with Aksenov et al. (2014), who observed enhanced *in vitro* responses when OBP ligands were presented with citrus volatiles. Interestingly, the antennal OBPs tested by Aksenov et al. (2014) reacted only to mixtures of citrus volatiles, not to individual volatiles. Exposure to test ligands 654, 717, 784, and 861 may have made the psyllids more receptive to the Cmac scent, and possibly to other close-range chemosensory, visual, and vibrational stimuli used by the psyllids to select host plants and located conspecifics. Another possibility is that, once the psyllids perceived these test ligands, they became stimulated to follow the concentration gradient of Cmac scent emanating from the vials.

### Ligand Inhibiting the Response of *D. citri*

In the Ligand 019 experiment, the Cmac scent treatment vials had numerically the fewest psyllids while the blank SPLAT vials had high aggregation levels. These results indicated that Ligand 019 either interfered with or inhibited the attraction and aggregation responses of *D*. *citri* to the Cmac scent. Since numerically more psyllids settled in vials with Ligand 019 than in Cmac scent, the ligand itself did not appear to be a repellent to *D*. *citri*; nonetheless, it influenced the behavioral response of the psyllids to the Cmac scent.

### OBP Ligands With No Apparent Effects on *D. citri*

Although there were overall treatment effects in the experiment with Ligand 937, there were no significant differences in psyllid settling levels between individual treatments. A numerically greater number of psyllids settling in the Cmac scent treatment vials than in the vials with blank SPLAT and the percentage of vials with ≥7 psyllids was significantly greater in vials with Cmac scent versus blank SPLAT. Whereas Ligand 937 induced high levels of probing, its effects on psyllid settling or aggregation behavior were not as obvious. Ligand 905 had no observable effect on psyllid settling response but the percentage of vials with ≥7 psyllids was significantly greater in vials with either Cmac scent or 9 mg Ligand 905 versus blank SPLAT.

### Conclusion and Possible Application

In the present study, the simultaneous presentation of a synthetic OBP ligand and a mixture of citrus volatiles influenced two behaviors in *D*. *citri*, namely, host plant attraction and aggregation on host plants. It seems likely that the psyllids perceived a test ligand and the Cmac scent volatiles within the confines of the incubators prior to their entry into the vials. Within the incubators, psyllids frequently moved up and down from the overhead lights to the vial caps; this would have given them the opportunity to come into contact with test ligand and the Cmac scent while outside the vials.

Interestingly, increased settling and aggregation levels were largely not observed in the vials with the test ligand treatments, and there was little effect of ligand concentration on psyllid response levels. This gives credence to the notions that the psyllids perceived the test ligands while in the incubator and that the test ligands themselves did not attract the psyllids. While it may seem intuitive that the psyllids perceived the ligands and citrus volatiles outside of the vials, our study did not include an evaluation of volatile emission levels from the SPLAT droplets, at the vial entrance holes, or within the incubator atmosphere. Therefore, we did not have empirical evidence that behaviorally meaningful ligand or citrus volatile concentrations were present outside of the vials, i.e., in the chamber atmosphere. In fact, our *a priori* assumption was that the greatest level of olfactory stimulation occurred when the psyllids perched at the entrance holes of the vials, where they would have been exposed to ligand concentrations representative of the vial interiors rather than the chamber atmosphere. We conjectured that the combination of visual and olfactory stimuli presented to the psyllids at the entrance holes would cause them to enter the vials. The results obtained instead suggest that it is more likely that the psyllids perceived an “average” level of ligand in the chamber atmosphere, which would have been a function of the total amount of volatilized ligand emitted from all of the ligand treatment vials, and once they perceived the ligand, they responded more strongly to the Cmac volatiles. Further studies using electrophysiological assays, the response of DCSAP4 and other psyllid OBPs to the different ligands, and/or more refined behavioral assays are needed to determine whether psyllids respond to the ligand and citrus volatiles concomitantly or if perception of the ligand increases psyllid response to the citrus volatiles. Lastly, if some of the ligands had low volatility, it may help explain why there was no behavioral effect observed in experiments where they were presented to the psyllids. This is another area for further investigation.

It is also curious that many vials had no psyllids in them, since each contained a young citrus shoot and these were expected to be highly attractive themselves to the psyllids. The numerous vials observed with no or only a single psyllid in them also gives support to the idea that a combinational or synergistic effect between the test ligand and Cmac scent occurred and that this interaction was an initial step leading to the development of aggregations within vials with Cmac scent. The synthetic ligands tested in this study could be good candidates to further examine the molecular underpinnings of *D*. *citri* perception of chemosensory cues.

In terms of application, these results indicate that the addition of some of these test ligands to mixtures of citrus volatiles could significantly promote the effectiveness of these mixtures as scent lures for *D*. *citri* in conjunction with monitoring traps or attract and kill devices. Ligand 019 might be effective at repelling psyllids. However, further work is needed with respect to evaluating the toxicology of the ligands and ensuring that they are safe to release in the environment, whether their performance in the field significantly elevates psyllid capture levels, and whether the additional cost of adding them to scent lures or trapping devices is justified in terms of enhanced psyllid capture level.

## Author Contributions

JP invented the settling assay, designed the experiments, supervised the execution of the project, conducted some of the statistical analysis, and wrote the manuscript. WM conducted the statistical analyses in the main, contributed to the “Statistical Analysis” and “Results” sections of the manuscript, and contributed to the development of the manuscript. RN contributed to the experimental design and statistical analyses. DW provided the synthetic ligands and contributed to the experimental design.

## Conflict of Interest Statement

The authors declare that the research was conducted in the absence of any commercial or financial relationships that could be construed as a potential conflict of interest.
