# Supplementary material for: Synthetic Ligands of Olfactory Binding Proteins Modulate Aggregation Response of Asian Citrus Psyllid in the Presence of Host-Plant Volatiles
Source: Front Plant Sci. 2018 Dec 20;9:1891. doi: 10.3389/fpls.2018.01891 (PMC6306466; doi:10.3389/fpls.2018.01891)

Supplemental Figure A1. Observed frequency distribution of the numbers of psyllids/vial compared to the Poisson distribution for each treatment in the control test without ligands. * = $P \leq 0.05$; ** = $P \leq 0.01$; *** = $P \leq 0.001$; chi-square test.

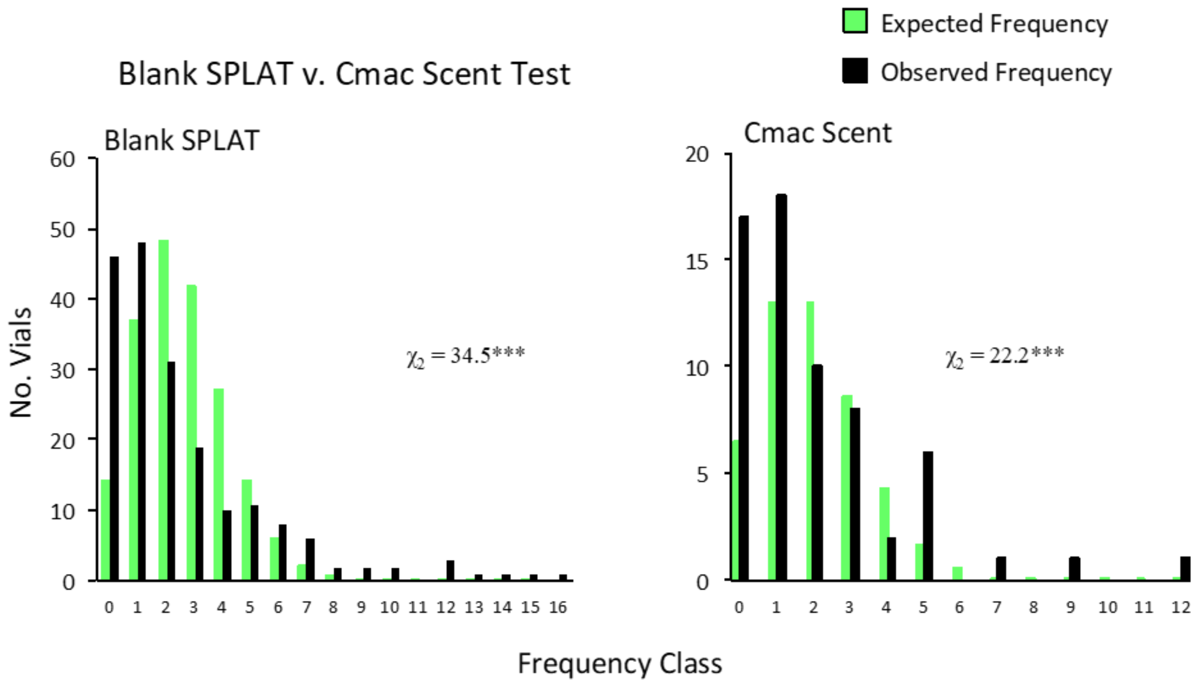

Supplemental Figure A2. Observed frequency distribution of the numbers of psyllids/vial compared to the Poisson distribution for each treatment in the Ligand 717 tests.
* = $P \leq 0.05$; ** = $P \leq 0.01$; *** = $P \leq 0.001$; chi-square test.

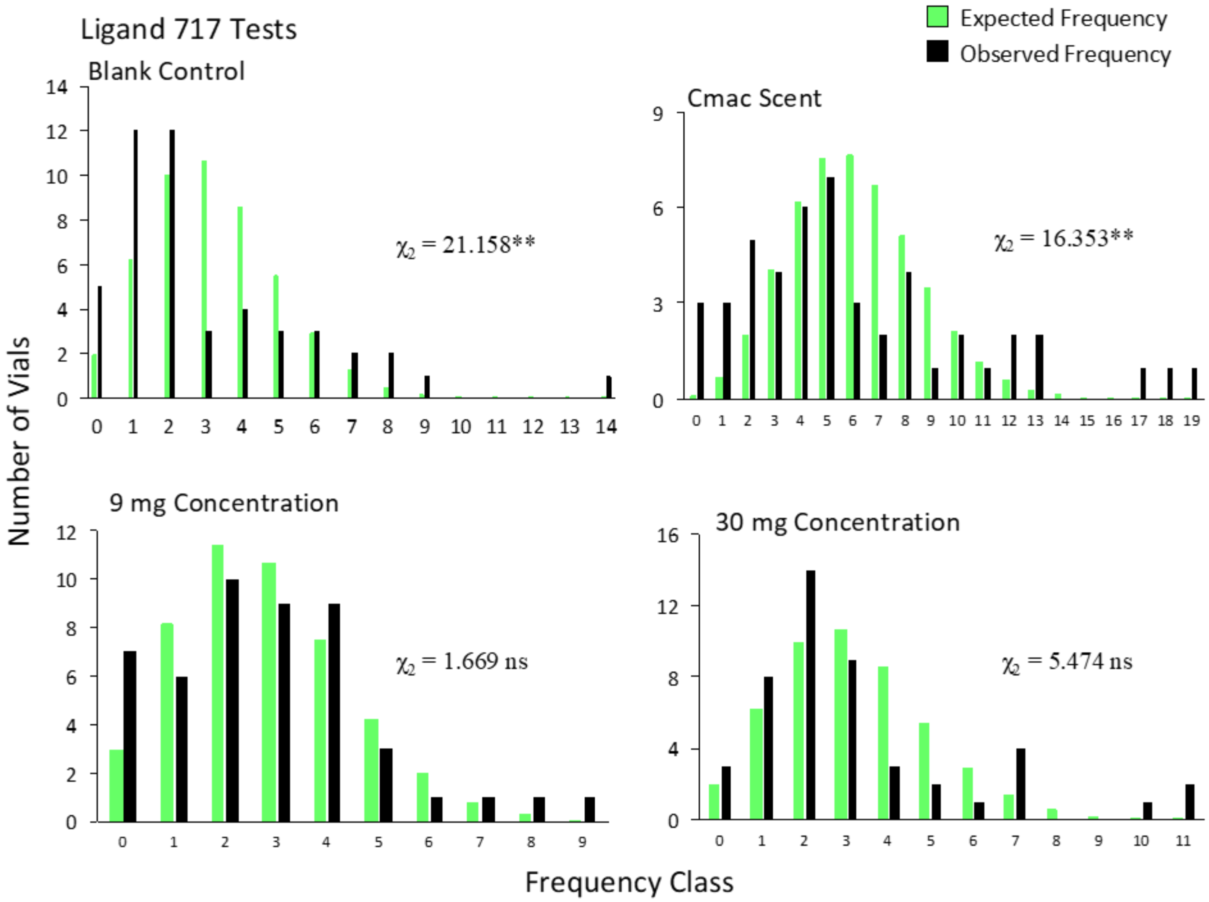

Supplemental Figure A3. Observed frequency distribution of the numbers of psyllids/vial compared to the Poisson distribution for each treatment in the Ligand 784 tests.
* = $P \leq 0.05$; ** = $P \leq 0.01$; *** = $P \leq 0.001$; chi-square test.

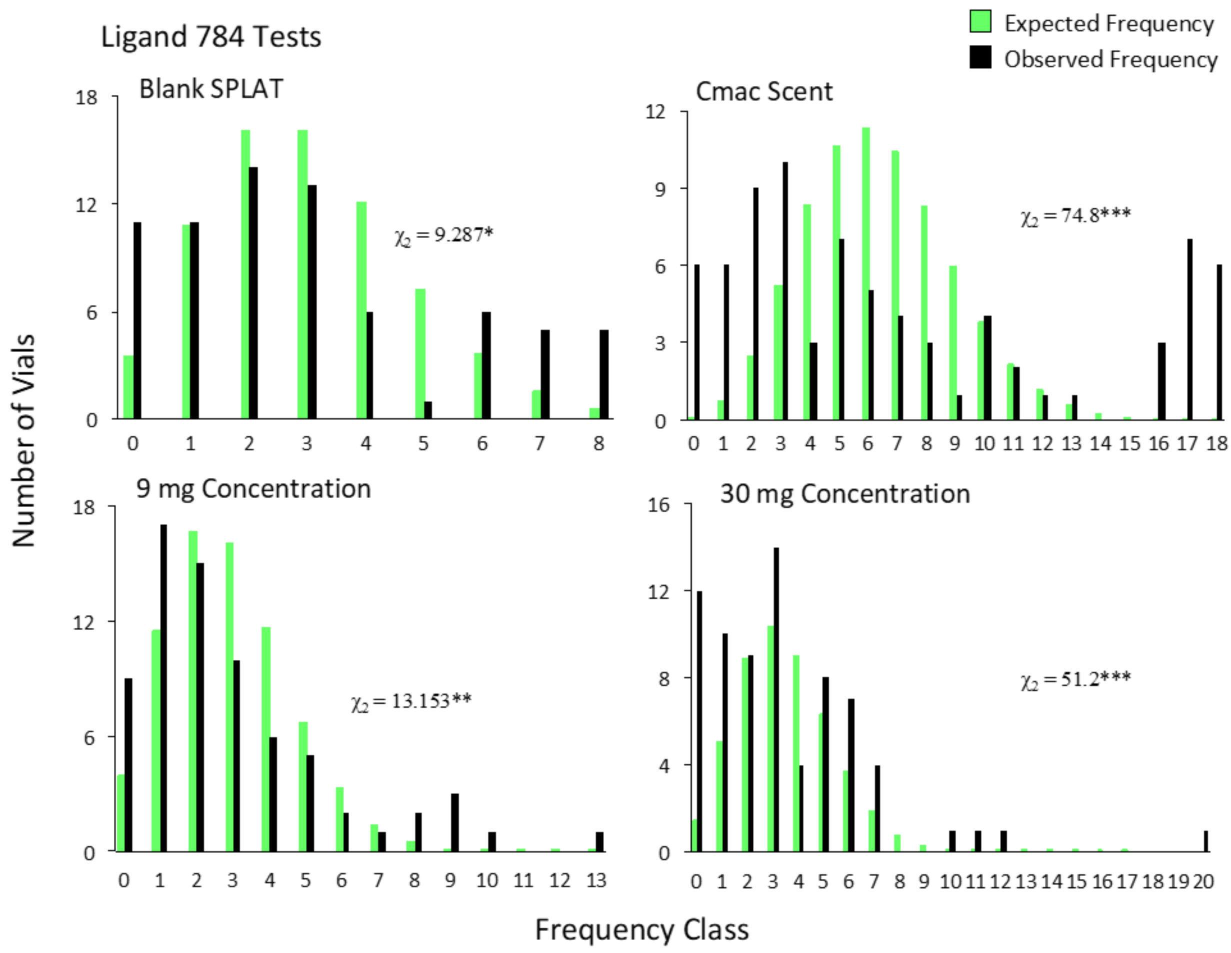

Supplemental Figure A4. Observed frequency distribution of the numbers of psyllids/vial compared to the Poisson distribution for each treatment in the Ligand 861 tests.
* = $P \leq 0.05$; ** = $P \leq 0.01$; *** = $P \leq 0.001$; chi-square test.

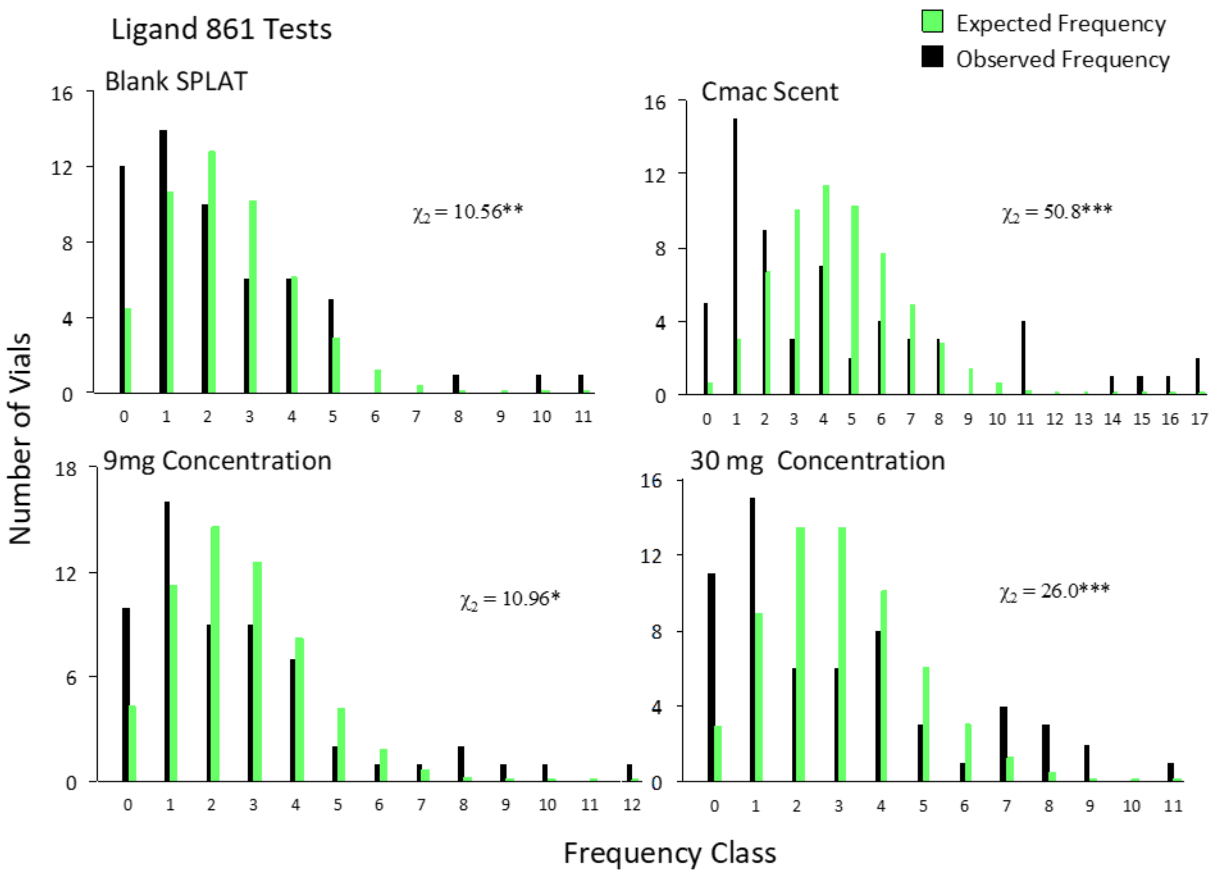

Supplemental Figure A5. Observed frequency distribution of the numbers of psyllids/vial compared to the Poisson distribution for each treatment in the Ligand 654 tests.
* = $P \leq 0.05$; ** = $P \leq 0.01$; *** = $P \leq 0.001$; chi-square test.

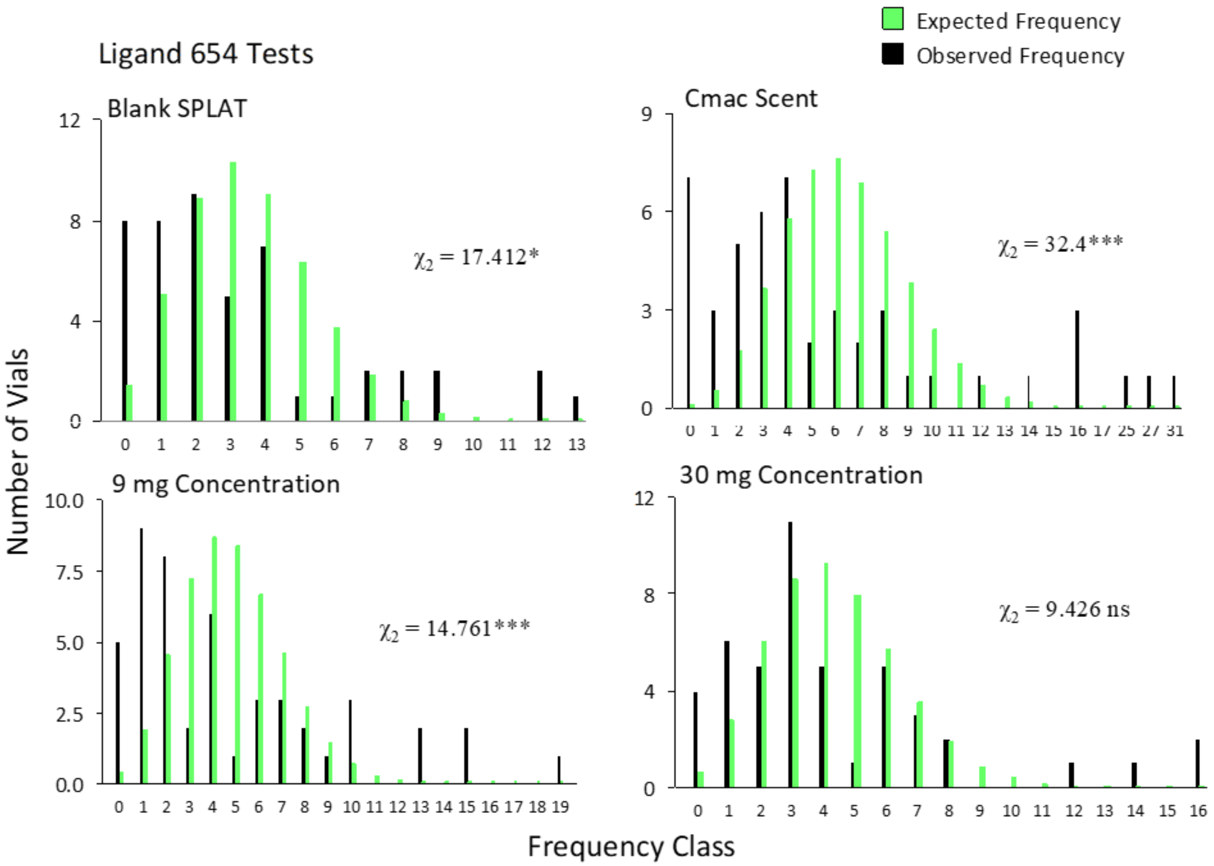

Supplemental Figure A6. Observed frequency distribution of the numbers of psyllids/vial compared to the Poisson distribution for each treatment in the Ligand 019 tests.
* = $P \leq 0.05$; ** = $P \leq 0.01$; *** = $P \leq 0.001$; chi-square test.

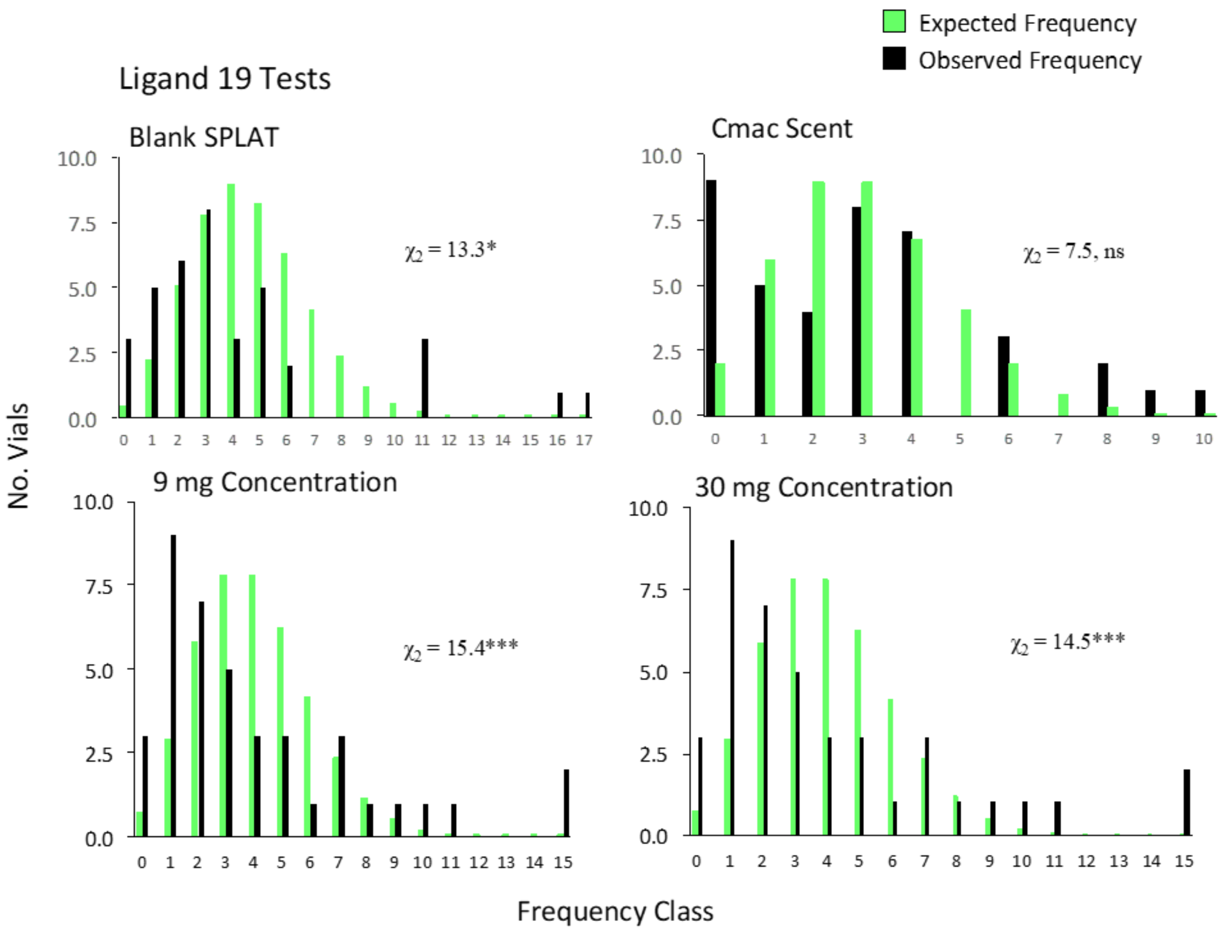

Supplementary Figure A7. Observed frequency distribution of the numbers of psyllids/vial compared to the Poisson distribution for each treatment in the Ligand 905 tests.
* = $P \leq 0.05$; ** = $P \leq 0.01$; *** = $P \leq 0.001$; chi-square test.

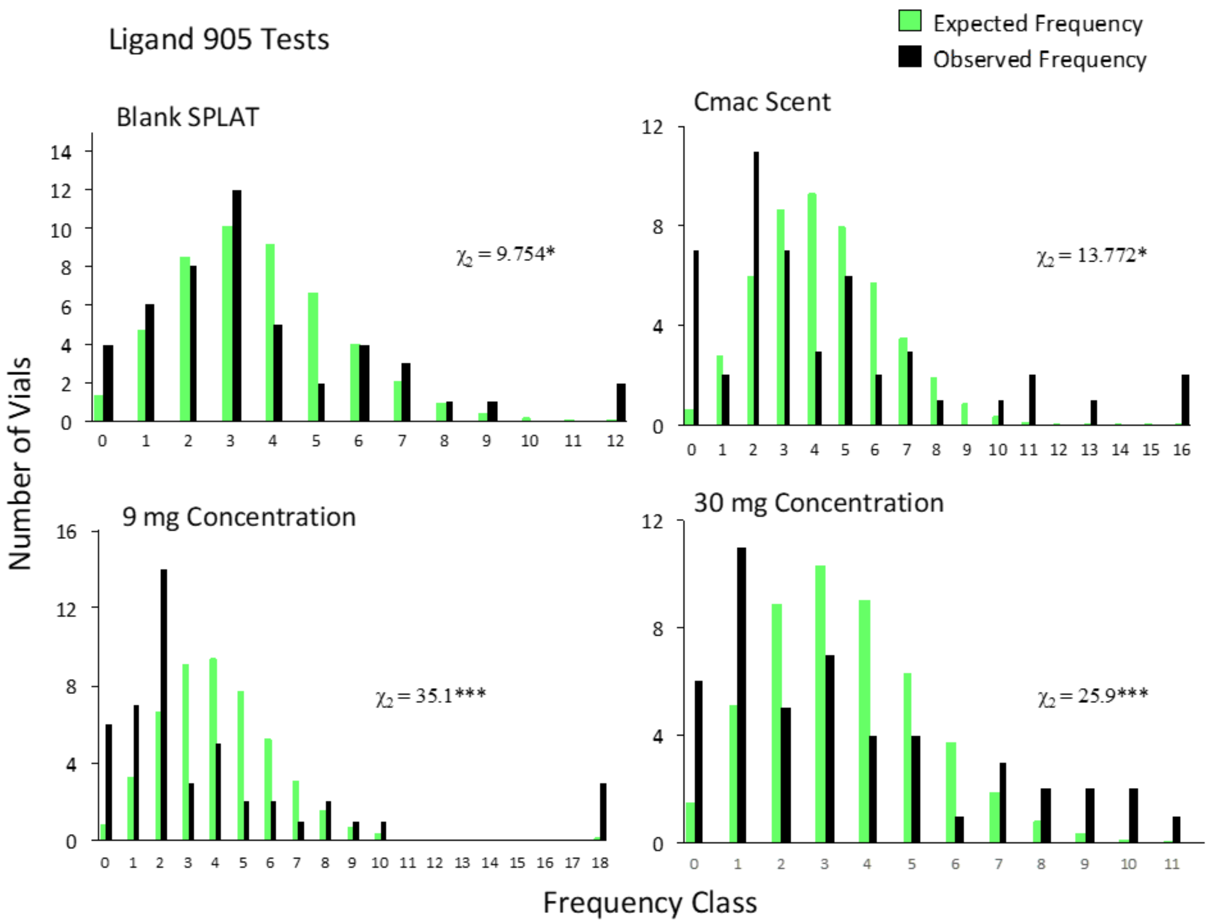

Supplemental Figure A8. Observed frequency distribution of the numbers of psyllids/vial compared to the Poisson distribution for each treatment in the Ligand 937 tests.
* = $P \leq 0.05$; ** = $P \leq 0.01$; *** = $P \leq 0.001$; chi-square test.

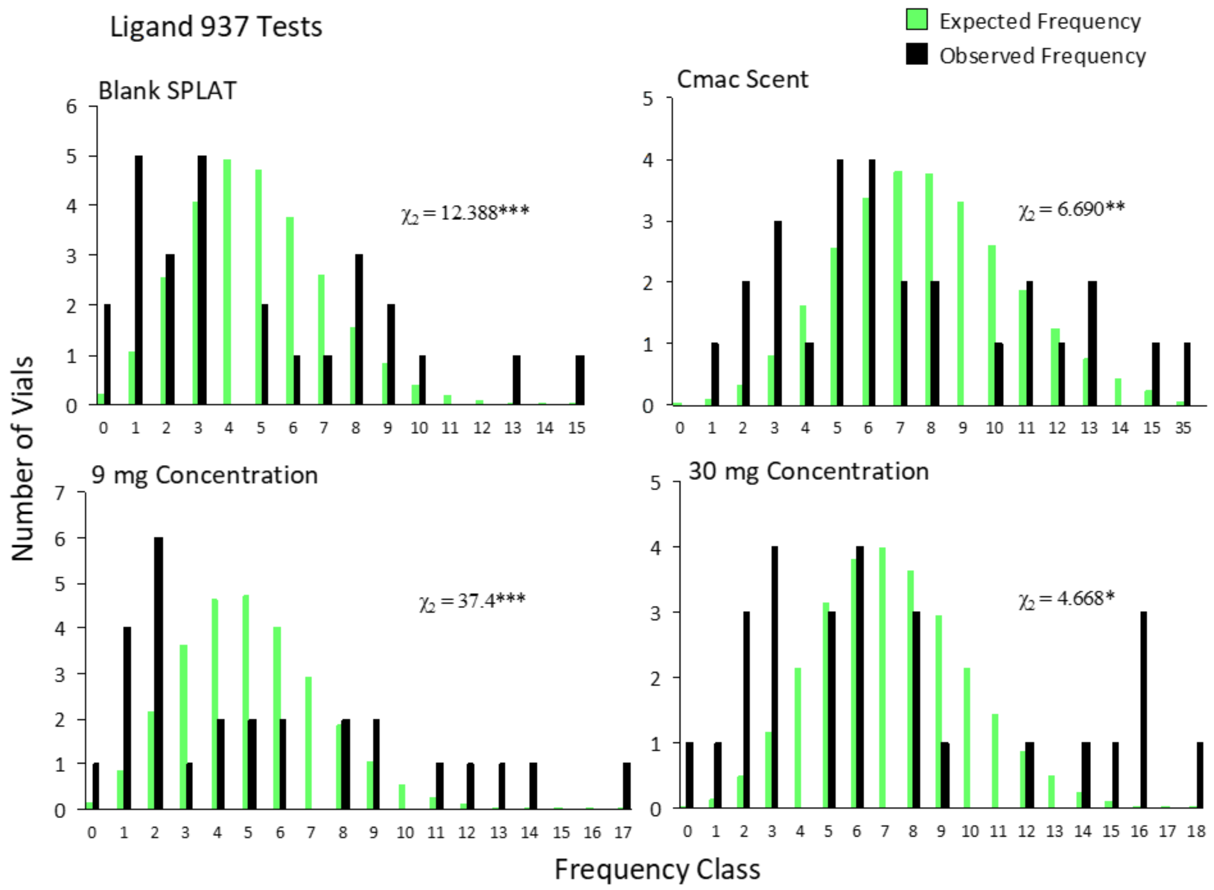

Supplement: Supplementary file 1 [file Data_Sheet_1.PDF]
